# Indication of intravaginal insemination for infertility treatment in couples with sexual dysfunction

**DOI:** 10.1002/rmb2.12376

**Published:** 2021-03-13

**Authors:** Hiroshi Kaseki, Satoshi Kaseki, Makiko Shimizu, Ayako Hayashi, Nobuhiko Suganuma

**Affiliations:** ^1^ Kaseki Ladies Clinic Ichinomiya Aichi Japan; ^2^ Department of Obstetrics and Gynecology Kariya Toyota General Hospital Kariya Aichi Japan; ^3^ Depatment of Nursing Aichi Medical University Nagakute Aichi Japan; ^4^ Department of Nursing Nagoya University of Arts and Sciences Nagoya Aichi Japan

**Keywords:** assisted reproductive technology, erectile dysfunction, infertility treatment, intravaginal insemination, sexual dysfunction

## Abstract

**Purpose:**

To analyze the usefulness of intravaginal insemination (IVI) for the infertility treatment in couples with sexual dysfunction before applying assisted reproductive technology (ART).

**Methods:**

Among 208 couples who presented sexual dysfunction, 144 couples underwent IVI procedures. The profiles of pregnant and non‐pregnant patients were compared.

**Results:**

Of 144 patients, 58 women conceived successfully (40.3% pregnancy rate). Between the pregnant and non‐pregnant cases, the husband's age and infertility period were significantly higher (*P* = .0104) and longer (*P* = .0027) in the unsuccessful cases than the successful ones. The husbands who could not impregnate had a significantly higher ratio of sperm abnormalities (*P* = .0048). Among the 57 successful cases who underwent IVI treatment, 38 (66.7%) patients became pregnant within 3 times of the procedure, while 48 (84.2%) patients conceived within 6 times.

**Conclusion:**

The authors can propose the following inclusion IVI criteria for couples with sexual dysfunction: (a) younger husband (36 years old or less) which may be most important, (b) infertility duration of 3 years or less, (c) normal sperm condition, and (d) IVI trial for 3 times (maximum of 6 times). Since IVI appears to be a simple, noninvasive, and inexpensive way for couples with sexual dysfunction, it can be attempted before ART application.

## Graphical abstract

Intravaginal insemination (IVI) is a useful method for the infertility treatment in couples with sexual dysfunction before ART application. The following inclusion criteria can be proposed; (a) younger husband (36 years old or less), (b) infertility duration of 3 years or less, (c) normal sperm condition, and (d) IVI trial up to 3 times (maximum of 6 times).

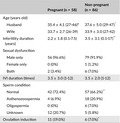

## 1 INTRODUCTION

Sexual dysfunction is a serious problem resulting in both sexual and reproductive issues. Male sexual dysfunction such as low libido, erectile dysfunction (ED), and premature or inhibited ejection, and female dysfunction such as inhibited sexual desire, inability to become aroused, anorgasmia, vaginismus, and dyspareunia, can result in unsatisfied sex or sexless situation. Epidemiological studies reveal that male sexual dysfunction has a prevalence of 5%‐30%,^1^, ^2^, ^3^ which shows that sexual conditions present not only medical but also social problems.^4^

Moreover, without insertion of penis into the vagina and/or no ejection of semen in the vagina, the sperm cannot be inseminated into the woman's reproductive organ, which causes infertility. Conversely, assisted reproductive technology (ART) has made it possible to conceive without coitus. Intrauterine insemination (IUI) is widely used as the treatment method for mild male factor and unexplained infertilities.^5^ In vitro fertilization (IVF) and a microinsemination of intracytoplasmic sperm injection (ICSI) are further applied; more than 465,000 babies were born in 2012 using these technologies throughout the world.^6^ Although ART is very effective as an infertility treatment, IUI can be painful when a speculum is inserted into the vagina and further, women are required to visit the clinic repeatedly. IVF and ICSI also involve burdensome processes such as ovarian stimulation by gonadotropins, frequent clinic visits to follow the follicle growth, and oocyte retrieval under anesthesia, In addition, IUI, IVF, and ICSI are all expensive with a cost of about 10 000‐30 000, 200 000‐500 000, and 300 000‐600 000 yen, respectively, without national health insurance coverage in Japan.

Contrarily, intravaginal insemination (IVI) as an artificial home vaginal insemination is a simple, noninvasive, and inexpensive method, which does not require frequent doctor visits. IVI can be performed personally at home in a private room, and does not impact marital activities. IVI may further be applied to treat couples with sexual dysfunction if semen can be obtained by the husband through masturbation. Pregnancy should be achieved without penile insertion into the vagina in an unconsummated marriage.

Although many studies regarding IVI on ejaculatory dysfunction have been reported, only a few studies on the effectiveness of IVI as an infertility treatment for couples with general sexual dysfunction, especially ED, are available. Our clinic, which is specialized in infertility management for more than 10 years, has helped many patients, including couples with sexual dysfunction. Thus, the authors analyzed the usefulness of IVI before applying ART and proposed the inclusion criteria of IVI as a treatment method for infertility.

## 2 PATIENTS AND METHODS

### 2.1 Patients

Between January 2009 and December 2018, 5034 couples first visited Kaseki Ladies Clinic for infertility treatment. Among these patients, 208 couples presented sexual dysfunction; 199 husbands indicated ED and/or inhibited ejection, and 20 wives had vaginismus and dyspareunia. Seventeen men had been treated with anti‐phosphodiesterase‐5 medicines such as sildenafil, vardenafil, and/or tadalafil. Some couples had received psychological sex counseling from specialists.

Other complications such as depression, hypertension, spinal cord injury (SCI), and cerebral infarction were observed in males, while among the wives, polycystic ovary syndrome, endometriosis, uterine myoma, ovarian cysts, and depression were present in 20, 7, 6, 4, and 3 cases, respectively. Fifty‐eight women had previously been pregnant and 47 had given birth. Four cases lost the ability to have sexual intercourse after delivery.

Inclusion criteria for the IVI treatment were as follows: (a) consent to undergo IVI treatment at home, (b) ability to collect sufficient volume of semen, and (c) an age of over 20 years for both husband and wife. Exclusion criteria were as follows: (a) non‐agreement for IVI, (b) inability to collect semen, (c) azoospermia, severe oligospermia, or evidential asthenozoospermia, and (d) absolute tubal or uterine factor infertility.

Any personal information in the clinical medical records used in this retrospective study was kept confidential, and all data were anonymized. Subsequently, the data sheet was transferred to the analyzer to sort out the data and analyze statistically. All these procedures were approved by the Ethical Committee at the Kaseki Ladies Clinic (R2‐01).

### 2.2 Intravaginal insemination procedure

After obtaining informed consent from the patients, 5‐10 sets of a sterile semen container (Multipurpose Beaker, 100 mL, Greiner Bio‐One International) and 1‐cc syringe (Terumo Corporation) were given to the couples. The IVI method was explained orally by the clinicians using a method sheet prepared by our clinic. The IVI procedure was performed at the time of ovulation, which was determined by transvaginal ultrasound imaging or by the couples themselves during the general menstrual cycle. When necessary, ovulation induction was performed using medicines such as clomiphene citrate (CC), human follicle‐stimulating hormone (FSH), or human menopausal gonadotropin (hMG). After the semen was collected in the container by masturbation, it was aspirated into the syringe and injected into the wife's vagina at home. These procedures were performed twice (up to 2 mL), and the wife then rested for about 15 minutes in the supine position. The patient was asked to return to our clinic when the pregnancy test was positive or all the sets of semen container and syringe were used. No troubles in IVI procedure were complained by the couples.

### 2.3 IUI and IVF/ICSI

ART was applied in the usual method at our clinic. IUI was performed using the swim‐up sperm. Ovarian stimulation with CC, FSH, or hMG, gonadotropin‐releasing hormone analog treatment, human chorionic gonadotropin injection, oocyte collection, sperm preparation, IVF, ICSI, and fresh or cryopreserved‐thawed embryo/blastocyst transfer were performed mainly according to the standard methods used in Japan.^7^

### 2.4 Statistical analysis

To compare between pregnant and non‐pregnant cases who underwent IVI, Student's *t* test, chi‐square test, or the Cox regression proportional hazard model was applied for statistical analysis using SPSS Statistics, version 25 (IBM Japan, Tokyo, Japan). A *P*‐value of <.05 was considered statistically significant.

## 3 RESULTS

### 3.1 Patient profiles

**FIGURE 1:**
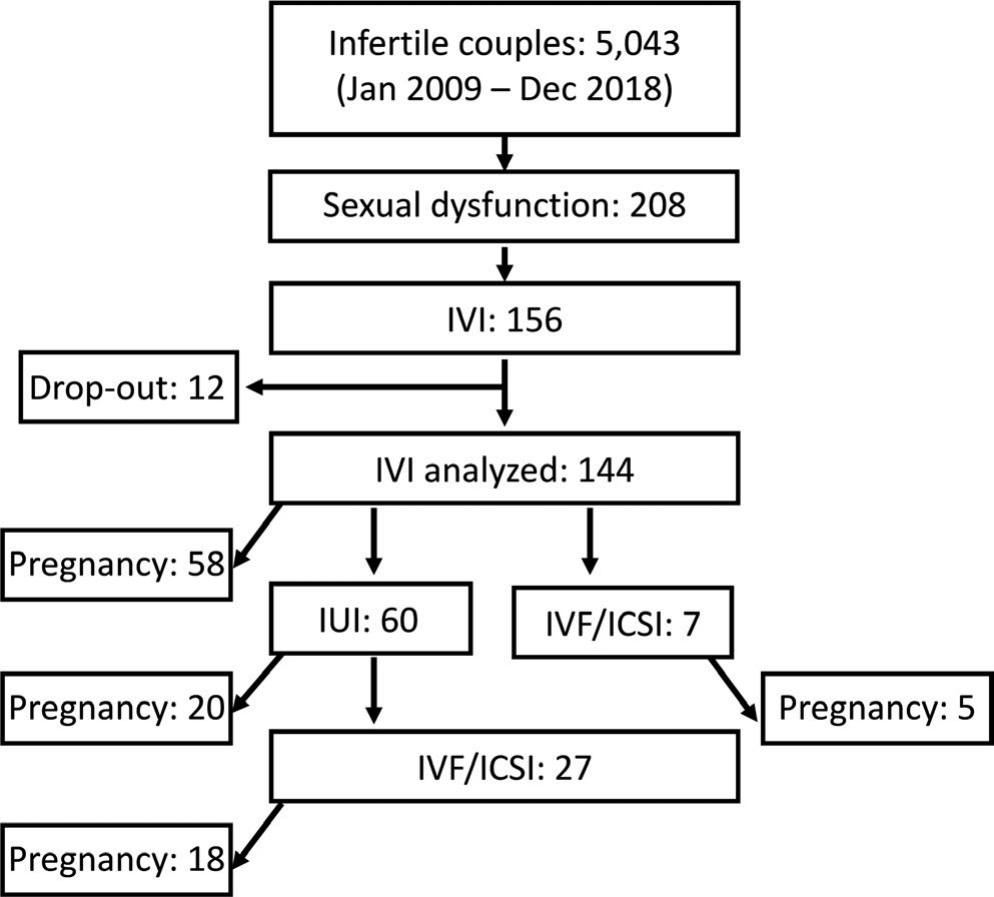
Treatment process of infertile couples with sexual dysfunction by intravaginal insemination (IVI) and other assisted reproductive therapies such as intrauterine insemination (IUI) and in vitro fertilization (IVF)/intracytoplasmic sperm injection (ICSI). The number of pregnant cases is further indicated after each treatment

Because 12 of the 156 couples who received IVI instruments did not visit our clinic again, the outcome of IVI treatment among the remaining 144 cases was analyzed (Figure 1). Results showed that 58 women conceived successfully as a pregnancy rate (PR) of 40.3%.

A comparison of the pregnant and non‐pregnant cases showed that the husband's age and infertility period were significantly higher (*P* =.0104) and longer (*P* =.0027) in the unsuccessful cases than the successful ones (Table 1). There was no difference in pregnancy achievement among the couples who had male factors, female factors, or both (*P* =.2526). The number of IVI performances was not different in the both groups (*P* =.9941). The husbands who could not impregnate had a significantly higher ratio of sperm abnormalities such as asthenozoospermia and oligospermia (*P* =.0048). Ovulation induction with CC, FSH, and/or hMG was performed significantly more in pregnant women (*P* =.0161).

**TABLE 1:** Profiles of couples resulted successful and unsuccessful outcomes by IVI

	Pregnant (n = 58)	Non‐pregnant (n = 86)
Age (years old)		
Husband	35.4 ± 4.1 (27‐46)^a^	37.6 ± 5.0 (29‐47)*
Wife	33.7 ± 2.7 (26‐39)	33.9 ± 3.0 (27‐42)
Infertility duration (years)	2.2 ± 1.8 (0.1‐7.5)	3.5 ± 3.1 (0.1‐17)**
Sexual dysfunction		
Male only	56 (96.6%)	79 (91.9%)
Female only	0 (0%)	1 (1.2%)
Both	2 (3.4%)	6 (7.0%)
IVI duration (times)	3.5 ± 3.0 (1‐12)	3.5 ± 2.0 (1‐12)
Sperm condition		
Normal	42 (72.4%)	57 (66.2%)**
Asthenozoospermia	4 (6.9%)	18 (20.9%)
Oligospermia	0 (0%)	6 (7.0%)
Unknown	12 (20.7%)	5 (5.8%)
Ovulation induction	11 (19.0%)	6 (7.0%)*

Abbreviation: IVI, intravaginal insemination.

^a^ Mean ± SD (range).

* *P* < 0.05.

** *P* < 0.01.

### 3.2 Number of IVI procedures until pregnancy

The number of IVI treatment performed until pregnancy in the successful cases is summarized in Table 2. In the 57 cases except an “unknown” one, 38 (66.7%) patients got pregnant within 3 times of performing the procedure, while 48 (84.2%) patients got pregnant within 6 times.

**TABLE 2:** IVI treatment duration until pregnancy (n = 58)

Duration (times)	Cases
1	20 (34.5%)
2	10 (17.2%)
3	8 (13.8%)
4	2 (3.4%)
5	4 (6.9%)
6	4 (6.9%)
7	3 (5.2%)
8	1 (1.7%)
9	1 (1.7%)
10	2 (3.4%)
11	0 (0.0%)
12	2 (3.4%)
Unknown	1 (1.7%)

### 3.3 Cox regression proportional hazard model

Analysis using the Cox regression proportional hazard model was performed. Because regression equation indicated as *P* =.003, the regression equation for each parameter was analyzed subsequently. As shown in Table 3, the hazard ratio of husband's age indicated a significant relation with the outcomes of IVI.

**TABLE 3:** Relation of each parameter to the IVI outcomes

	Hazard ratio	95% confidence interval	*P*‐value
Husband's age	0.916	0.839‐0.999	.047
Wife's age	0.987	0.851‐1.144	.863
Sexual dysfunction	1.872	0.253‐13.872	.540
IVI duration	0.863	0.733‐1.105	.075
Sperm condition	2.454	0.837‐7.191	.102
Ovulation induction	0.588	0.279‐1.240	.163

Note

Cox regression proportional hazard model was applied.

Abbreviation: IVI, intravaginal insemination.

### 3.4 Outcomes by other ART treatments

The couples who could not become pregnant by IVI were treated using other ARTs (Figure 1). IUI was performed in 60 patients, which resulted in 20 pregnancies (33.3% PR). Twenty‐five of the 34 cases treated with IVF/ICSI after IUI or directly became pregnant (73.5% PR). Moreover, in the cases with sexual dysfunction who did not receive IVI procedure, 18 couples underwent IUI of which 4 conceived, while 10 couples underwent IVF/ICSI of which one woman became pregnant. Altogether, in this series, 58 of 144 (40.3% PR), 24 of 78 (30.8% PR), and 26 of 44 (59.1% PR) couples with sexual dysfunction could get pregnant by IVI, IUI, and IVF/ICSI, respectively.

During 2009‐2018, a total number of 8,334 IUI in 3,132 couples with infertility of other causes were performed at our clinic. Five hundred and eighty women became pregnant as 18.7% PR/case and 7.0% PR/performance. In this period, 3, 956 IVF/ICSI cycles in 2,973 patients were also carried out. The PRs/cycle of 33.0% (343/1,039) and 34.6% (1883/5441) were obtained in fresh and cryopreserved‐thawed embryo transfers, respectively. These results indicated that effectiveness of the ART in couples with sexual dysfunction can be comparable with other infertile patients.

## 4 DISCUSSION

For a long time, IVI has been applied to treat ejaculatory dysfunction.^8^ Kathiresan et al^9^ reported that IVI was performed in 45 couples in whom the husbands had SCI; 17 of them achieved 20 pregnancies (37.8% PR). Sønksen et al^10^ indicated that 60 of the 140 couples (42.9% PR) in whom the husband suffered SCI achieved 82 pregnancies by IVI at home, and 72 of the pregnancies resulted in live births with the delivery of 73 healthy babies. In summary, IVI had 25%‐70% PR in couples in whom the male partner had SCI.^11^

However, IVI has not been widely applied in couples with sexual dysfunction. In our study, a PR of 40.3% by IVI was observed in 144 cases with sexual dysfunction, which is comparable to the results obtained among SCI patients. Banerjee and Singla^12^ also demonstrated that 29 women among 55 couples with an unconsummated marriage could conceive by IVI (52.7% PR). Their results showed that the younger husbands aged 36 years or less had better success rates, similar to our results. These observations demonstrated that IVI might be a useful initial method for younger husbands in couples with sexual dysfunction.

Our results showed that a longer infertile duration resulted in a reduced conception rate, similar to results from other studies on IUI. Zadehmodarres et al^13^ stated that the PR in a group with an infertility duration of 3 years or less was significantly higher than that in a group with a longer period of infertility. Kamath et al^14^ indicated that the duration of infertility was significantly associated with the chance of success (5.36 vs 6.71 years, *P* =.032). Ghaffari et al^15^ further demonstrated that an infertility duration of 4 years or less was associated with a significantly higher pregnancy rate (OR: 1.5, CI: 1.1‐2.2, *P* =.01) in 803 infertile couples. Although the duration lengths were different in each study, earlier insemination resulted in successful outcomes.

Sperm condition is another important aspect of IVI treatment. When sperm examination gives poor results, ART should be immediately recommended. As indicated in the results, IUI and IVF/ICSI showed a very high success rate even in couples with sexual dysfunction. In the present study, successful IVI was observed among approximately one‐third of the couples at the first trial and in two‐third within 3 trials. More than 80% of the patients could conceive in less than 6 trials. Indeterminately prolonged IVI therapy may not be beneficial for the patients.

Although the scientific data regarding IVI effectiveness are not adequate, this method may be widely used because many types of commercial kits are available via online shopping sites. Many people are speculated to utilize those kits at home (called the “syringe method” in Japan), and the couples only visited clinics when the procedure ended in failure. Certainly, there are no problems in attempting IVI personally; however, insufficient processing may prolong the infertility period. In our study, the pregnant group with IVI demonstrated a higher ratio of ovulation induction than the non‐pregnant group, which shows that the detailed detection of ovulation timing by ultrasound imaging may be useful.

Because our study was performed at a private clinic, the number of cases and the therapeutic system might be insufficient. More scientific data, including a commercial base, should be represented to evaluate the effectiveness of IVI at home for couples with sexual dysfunction. Moreover, sexual dysfunction managed in the present study mainly consisted of male factors. The effectiveness of IVI for female sexual dysfunction should be analyzed using more such cases in the future research.

In conclusion, the authors propose the following inclusion criteria of IVI as an infertility treatment for couples with sexual dysfunction: (a) younger husband (36 years old or less), (b) infertility duration of 3 years or less, (c) normal sperm condition, and (d) IVI trial up to 3 times (maximum of 6 times). Among these proposals, husband's age may be the most important factor for the IVI outcomes though the reason will be able to be revealed in further study. IVI at home seems to be a simple, noninvasive, and inexpensive way for couples with sexual dysfunction to get pregnant; it can hence be attempted before IUI and/or IVF/ICSI are applied.

## CONFLICT OF INTEREST

The authors report no declarations of interest.

## ETHICAL CONSIDERATION

The protocol for the research project including human subjects has been approved by a suitably constituted Ethics Committee.
